# Dementia, home care and institutionalisation from hospitals in older people

**DOI:** 10.1007/s10433-018-0493-0

**Published:** 2018-11-28

**Authors:** Feifei Bu, Alasdair Rutherford

**Affiliations:** Faculty of Social Sciences, University of Stirling, Stirling, UK

**Keywords:** Institutionalisation, Hospital discharge, Dementia, Home care

## Abstract

This study examines the relationship between dementia and institutionalisation directly after hospital discharges in older people and explores potential mediators of the association. Our analyses are based on linked Scottish administrative health and social care data, including 79,983 hospital stays for 43,753 patients aged 65 or over. Our results show that dementia patients are more likely to be discharged to care institutions compared with non-dementia patients (odds ratio = 17). Further analyses reveal that this can be partially explained by the fact that dementia patients are more likely to have injury-related admissions and long hospital stays. This indirect effect accounts for around 6% of the total effect. We also investigate the effect of receiving home care, finding no evidence that it influences the likelihood of institutionalisation from hospitals in older people.

## Introduction

The last three decades have witnessed a rapid increase in the older populations of most developed countries, which is likely to continue or escalate over the next a few decades. In Scotland, for example, it is projected the number of older people aged 65 or over will increase by 53% from 2014 to 2039 and those aged 80 or over will be doubled over the same period of time (National Records of Scotland 2015). There are two main causes of the population ageing: the increase in life expectancy and the decrease of fertility rate (Lutz et al. 2008). Thanks to the improvement of living standards and health services, people can generally live longer but they may also spend a longer time in a state of illness and dependency as the increase in healthy life expectancy does not catch up with the increase in life expectancy (Public Health Information for Scotland 2015). Therefore, there will be an increasing demand for both informal and formal care. Moreover, a decreasing fertility rate means that fewer adult children will be available to provide care for their older parents, which will impose extra strains on the formal care system when informal care is unavailable.

Formal care can be provided in either community or institutional settings which can be referred to as community care and institutional care, respectively. In this paper we use the term ‘institutionalisation’ to refer to receiving institutional care in a long-term residential setting, the predominant form of which in Scotland is living in a care home. In the Scottish context, community care is similar to what we here call ‘home care’. This is a form of social care service provided in the recipient’s own home, and which includes personal care, routine household tasks, respite care, overnight live-in services. However, it is not limited to home care. Other types of care services, for example, community alarm, telecare, meals-on-wheels and self-directed support, could also be counted as community care. Community care has an advantage over institutional care in that it allows older people to live independently in their own homes, to maintain their social network and to enjoy a higher quality of life. Perhaps more importantly for policymakers and service providers, there is evidence that the cost of community care is significantly lower than institutional care even after accounting for the hidden cost of informal care providers (Chappell et al. 2004; Hollander and Chappell 2007). Therefore, there has been a policy initiative to prevent or delay institutionalisation and to support older people to live at home for as long as possible.

In this study, we will focus on institutionalisation from hospitals: older people who were admitted to hospital from private households then being discharged to care institutions after hospital admission. This is of particular interest as hospital discharges are the major source of admission for long-stay residences in care institutions for older people, at least in Scotland. Based on the Scottish Care Home Census, during 2014/2015 around 47% of care home residents were admitted from hospitals, in contrast to 31% from private homes; and there seems to be an upward trend in the share of care home admissions from hospitals in recent years (ISD Scotland 2015). Therefore, an investigation of care institution admission from hospitals will lead to a better understanding of the institutionalisation of older people in general.

In the literature, a number of predictors for the institutionalisation of older people have been identified, such as age, gender, ethnicity, socioeconomic status, civil status, living arrangement, social network, caregivers’ characteristics, health status, number of prescriptions, length of hospital stay, functional or cognitive impairment (e.g. Agüero-Torres et al. 2001; Andel et al. 2007; Yaffe et al. 2002). Some of these predictors are controversial, but it is widely accepted that dementia is one of the most, if not the most, important predictors for the institutionalisation of older people (Luppa et al. 2009). For instance, in a study using Swedish data, dementia accounted for 61% of the population attributable risk percentage of institutionalisation (Agüero-Torres et al. 2001). In Scotland, it was reported that in 2015 61% of long-stay residents in care homes were living with dementia, which had increased by more than a third since 2006 (ISD Scotland 2015). Although the impact of dementia on institutionalisation has already been well documented, its underlying mechanisms rarely come under scrutiny. This paper aims to explore mediators which further explain the difference in hospital discharge to care institutions between older people with and without a dementia diagnosis. We hypothesise that the relationship between dementia and hospital discharge to care institutions is partially mediated by admission due to injury and long hospital stays.

The predictors of institutionalisation, including dementia, have been widely examined in the literature, but we know little about the effective interventions that reduce the risk of institutionalisation, especially the role of receiving home care in preventing or delaying institutionalisation for older people. This could have valuable implications for service providers and policy makers, which is another main objective of the present study.

## Data and method

### Data and variables

Our data come from the Linked Health and Social Care Data (2010/11) which were developed by the Scottish Government, in collaboration with the Information Services Division (ISD) Scotland, Scottish health boards and local authorities (LAs). The linked datasets incorporate the information on hospital admission, prescription, mortality and social care. The data linkage has been carried out for five LAs, namely City of Edinburgh, Clackmannanshire, South Ayrshire, South Lanarkshire and Stirling. In this paper, we have excluded patients who are under 65 given only older people aged 65 or over are eligible for free personal care provided by LAs in Scotland. In our final dataset, a total number of 79,983 hospital stays were recorded for 43,753 patients.

The information on institutionalisation from hospitals is derived from the Scottish Morbidity Record (SMR) in the linked data. The SMR covers all patients who were admitted to NHS hospitals (including contracted NHS beds in non-NHS institutions) receiving care in the general or acute specialties between 1 April 2010 and 31 March 2011. The base unit of observation in the SMR is the so-called finished consultant episode, a period of care that a patient receives from a particular consultant. Based on these episodes, we have derived the continuous inpatient stay (CIS) which is an unbroken period of time that a patient spends as an inpatient. Then, using the information on discharge destination at the end of the CIS, we are able to tell whether a patient was discharged to an institution, including care homes and other unspecified institutions. According to the data assessment report (ISD Scotland 2012), the finely detailed discharge destination is relatively poorly coded with an accuracy rate of 67% across Scotland. However, most of the errors (more than 70%) occurred in the sub-dimension of data coding, whereas the broader first-digit coding is accurate. For example, discharge to a care home may be incorrectly coded as ‘discharge to other institutions’. This has little influence on our analysis as it is not our aim to distinguish between different types of care institutions.

An individual is defined as living with dementia if he or she has at least one SMR (either general/acute or mental health specialties) record with a dementia diagnosis in any condition field, or has any anti-dementia drug in their prescription records from 1981 when the earliest data are available to 31 March 2011. Following this principle, there are around 6% of individuals who are defined as dementia patients in our final dataset. This is reasonably consistent with the estimated prevalence in the UK, 7% (Prince et al. 2014), as it includes people who are undiagnosed and who have not been admitted to hospitals within the observational period. The potential mediators tested are ‘admission reason’ and ‘length of stay’, both of which are treated as binary variables. The former is defined as whether a patient is admitted due to injury; the latter is defined as whether a patient stays in a hospital for longer than 7 days. Without a clear convention for long stays in the literature, this definition is based on clinical advice and methodological judgements (23% of patients having a stay longer than 7 days). The home care indicator is derived from the home care census that was conducted in the last week in March 2010, meaning that this is a snapshot measure taken prior to hospital admissions for most patients. A binary variable records whether the patient received any form of social care within their home in the reference week.

Apart from the main variables of interest, we control for individuals’ general health condition using the Charlson Comorbidity Index (CCI) (Charlson et al. 1987). This is a well-validated tool to control for confounding effects or to be used as a predictor of future outcomes in clinical research (D’Hoore et al. 1996; Roffman et al. 2016). In addition, a measure on dependency level is also included, measured by the indicator of relative need (IoRN), which is commonly used to assess the relative dependency of older people in Scotland. It was originally developed to facilitate decision-making in service providing. The score is derived based on a questionnaire that covers assessments of activities of daily living, food and drink preparation, mental health and behavioural issues. The original IoRN score has nine categories, which we have re-categorised into two groups, high versus low dependency. In principle, only people who have been assessed for social care services by their local authorities will have a valid IoRN score. Those who have never applied for social care service are treated as a separate category. Moreover, not every LA used IoRN as an assessment tool or provided information on it, leading to a substantial amount of missing data on this measure which are coded as a distinct category.

Another control variable is the Scottish Index of Multiple Deprivation (SIMD), an official measure used by the Scottish Government to identify small area concentrations of deprivation across Scotland. It is developed by incorporating seven different domains of deprivation and combining them into a single index which ranks each small area from 1 (most deprived) to over 6000 (least deprived) (Office of the Chief Statistician 2009). In this study, the SIMD is used as quintile measure with five ranked groups. Moreover, we also control for living arrangement, age at first admission and gender.

Among all variables, some of them are time-varying as people could be admitted to a hospital for multiple times. These variables include hospital admission source, admission reason, length of stay, living arrangement and discharge destination. The rest of the variables, for example, age, home care and dependency (IoRN scores), are treated as time-invariant because either they do not (or rarely) change over time by definition, or they were observed only once within the follow-up period.

### Method

Given the data we have, the independence assumption is violated as multiple hospital stays of the same patient will have correlated residuals. To relax this assumption, we employ random effects models here. Logit link functions are used as our outcome variable, and mediators are treated as binary.

1a $$\begin{aligned} {\mathrm{Logit}}[Pr(M_{ij}=1)]&=\beta _{10} + \beta _{11}d_{i} + \beta _{12}x_{ij} + \beta _{13}x_{i} + \mu _i \end{aligned}$$

1b $$\begin{aligned} {\mathrm{Logit}}[Pr(Y_{ij}=1)]&=\beta _{20} + \beta _{21}d_{i} + \beta _{22}x_{ij} + \beta _{23}x_{i} + \mu _i \end{aligned}$$

1c $$\begin{aligned} {\mathrm{Logit}}[Pr(Y_{ij}=1)]&=\beta _{30} + \beta _{31}d_{i} + \beta _{32}x_{ij}\nonumber \\&+\, \beta _{33}x_{i} + \beta _{34}M_{ij} + \mu _i \end{aligned}$$

To establish mediation effects, there are four conditions that need to be met (Baron and Kenny 1986; Judd and Kenny 1981; MacKinnon and Dwyer 1993). First of all, there must be an effect of the dementia variable on the mediators. As shown in Equation 1a, $M_{ij}$ represents the hypothesised mediators: hospital admission due to injury and long hospital stays. Both of these variables are time-varying. Individual patients are indicated by letter *i*, and the number of admissions is indicated by *j*. $d_{i}$ is our main variable of interest, dementia diagnosis, which is time-invariant. $x_{ij}$ represents a vector of time-varying control variables, for example, admission source, and CCI. The vector of time-invariant control variables is denoted by $x_i$, including age, gender, IoRN score and another variable of interest, home care. $\mu _i$ is the residual term, which we assume is not correlated with any of our explanatory variables.

Apart from an effect on the mediators, dementia must also have a significant influence on the outcome variable, discharge to care institutions, denoted by $Y_{ij}$ in Equation 1b. This is the second condition. $\beta _{21}$ is the total effect of dementia on discharge to care institutions. A third condition is that there must be an effect of the mediators on the outcome variable controlling for dementia (see Equation 1c). That is the coefficient, $\beta _{34}$, significantly differs from 0. $\beta _{31}$ is the direct effect of dementia on discharge to care institutions. Lastly, there must be a significant reduction in the magnitude of the dementia variable after controlling for the mediators. In linear models, the indirect effect is the difference between total effect and the direct effect ($\beta _{21}-\beta _{31}$). However, this is not the case in logit models as a variable has a different scale as a predictor than when it is used as an outcome variable in logit models. To test the significance of the indirect effect, we use the method developed by Karlson et al. (2012). For more detail on this method and its implementation, see also Karlson and Holm (2011) and Kohler et al. (2011). All of our models are fitted in Stata 14.

## Results

### Descriptive analysis

Table 1 shows the descriptive statistics by dementia diagnosis for a range of variables. There is a striking difference in discharge to care institutions between patients with and without a dementia diagnosis: more than 16% of dementia patients were discharged to care homes in contrast to only around 1% for patients without dementia. The figures also suggest that dementia patients are more likely to be admitted to hospitals due to injury-related reasons and to have long hospital stays. In addition, dementia patients seem to be more likely to use home care services: more than 32% of dementia patients received home care services compared with around 9% for non-dementia patients. Although a higher percentage of dementia patients were discharged to care institutions, we note that a higher percentage of dementia patients were admitted from institutional settings to begin with. Moreover, they are generally older and tend to have worse health conditions and a higher level of dependency. These descriptive statistics illustrate the importance of controlling for potential confounding variables in the assessment of the relationship between dementia and hospital discharge to care institutions.

**Table 1:** Descriptive statistics by dementia diagnosis

	Patient with dementia	Patient without dementia
Discharged to a care institution	16.22%	1.06%
Admitted due to injury	13.06%	4.42%
Long hospital stay (> 7 days)	47.31%	21.63%
Live alone prior to admission	2.84%	2.56%
Admitted from a private residence	85.70%	96.39%
Number of stays (n)	5358	74,615
Receive home care	32.82%	9.46%
Female	64.90%	55.85%
Age at first admission (mean)	83.09	76.30
IoRN score		
Low independence	4.52%	1.55%
High independence	4.07%	4.11%
Missing code	20.07%	13.90%
Not applicable	71.34%	80.44%
CCI		
Worst health	14.31%	7.92%
2	19.23%	11.52%
3	42.88%	15.99%
Best health	23.58%	64.57%
SIMD		
Most deprived	15.74%	14.94%
2	24.40%	22.91%
3	16.27%	18.90%
4	16.92%	16.29%
Least deprived	26.68%	26.29%
LA		
City of Edinburgh	48.50%	36.84%
Clackmannanshire	2.67%	4.16%
South Ayrshire	16.23%	15.89%
South Lanarkshire	27.20%	34.74%
Stirling	5.40%	8.36%
Number of patients (*N*)	3074	40,674

* IoRN* indicator of relative need;* CCI* Charlson Comorbidity Index;* SIMD* Index of multiple deprivation;* LA* local authority

### Mediation effects

We firstly examine the relationship between dementia diagnosis and our hypothesised mediators. Table 2 reports the random effect coefficient estimates of being admitted to hospital due to injury and long hospital stays, respectively. It is clear that older people living with dementia are significantly more likely to be admitted to hospital due to injury-related reasons in contrast to non-injury admissions, even after controlling for potential confounding variables. In addition, they are also more likely to have long hospital stays compared with other people without dementia.

**Table 2:** Impacts on hospital admission due to injury and long hospital stay (>7 days), N = 79,983 (43,753)

	Injury (vs. non-injury)		Long (vs. short) stay	
	Coeff.	SE	Coeff.	SE
Dementia patient	0.97***	(0.07)	0.41***	(0.05)
Receive home care in 2010	− 0.00	(0.07)	0.73***	(0.04)
Living alone prior to admission	1.13***	(0.10)	0.75***	(0.06)
Admitted from private residence	− 0.95***	(0.08)	− 0.79***	(0.05)
Female	0.68***	(0.05)	0.15***	(0.03)
Age^a^				
75–84	0.48***	(0.06)	0.64***	(0.03)
85 or over	1.13***	(0.07)	1.25***	(0.04)
IORN score^a^				
A–D	0.11	(0.17)	− 0.42***	(0.10)
0	− 0.09	(0.15)	− 0.36***	(0.09)
Missing	− 0.66***	(0.14)	− 1.06***	(0.09)
SIMD^a^				
2	− 0.01	(0.08)	0.03	(0.04)
3	0.02	(0.08)	− 0.02	(0.04)
4	− 0.04	(0.08)	− 0.07	(0.05)
Least deprived	0.00	(0.07)	− 0.15**	(0.04)
CCI^a^				
2			− 0.58***	(0.04)
3			− 0.60***	(0.04)
least severe			− 1.92***	(0.04)
LA^a^				
Clackmannanshire	− 0.42***	(0.13)	0.01	(0.07)
South Ayrshire	− 0.42***	(0.07)	− 0.61***	(0.04)
South Lanarkshire	− 0.62***	(0.06)	− 0.16***	(0.03)
Stirling	− 0.36***	(0.09)	− 0.12*	(0.05)
Constant	− 3.37***	(0.20)	0.51***	(0.04)
$\sigma _u$	1.82***	(0.07)	1.29***	(0.02)

*$$p<0.05$$, **$$p<0.01$$, ***$$p<0.001$$

^a^The reference category of the age variable is 65–74; the reference category for IORN is the most dependent; for SIMD is the most deprived; for CCI is the most severe; and the reference category for LA is the City of Edinburgh

Table 3 reports the coefficient estimates of institutionalisation after hospital admission. Model I includes dementia diagnosis as the only predictor. We see dementia patients are much more likely to be institutionalised compared with non-dementia patients (odds ratio = 141). However, similar to the descriptive analysis (Table 1), this estimate is likely to be confounded by other variables, for example, age, admission source, general health condition and independence level. Therefore, in Model II we have included a set of control variables, including home care. After controlling for these variables, dementia remains to a significant predictor of institutionalisation, but the magnitude of the coefficient has shrunk substantially. After controlling for potential confounding variables, the odds of being discharged to a care home for dementia patients are 17 times higher than that of non-dementia patients.

**Table 3:** Results from random effect logistic models on discharge to care institutions, N = 79,983 (43,753)

	Model I		Model II		Model III	
	Coeff.	SE	Coeff.	SE	Coeff.	SE
Dementia patient	4.95***	(0.13)	2.86***	(0.12)	2.80***	(0.12)
Receive home care in 2010			0.80***	(0.11)	0.57***	(0.11)
Living alone prior to admission			1.12***	(0.22)	0.93***	(0.22)
Admitted from private residence			− 2.72***	(0.10)	− 2.67***	(0.10)
Female			0.23**	(0.08)	0.17	(0.09)
Age^a^						
75–84			0.88***	(0.11)	0.75***	(0.12)
85 or over			2.15***	(0.13)	1.91***	(0.13)
IORN^a^						
*A*–*D*			− 1.00*	(0.46)	− 0.95*	(0.46)
0			0.08	(0.33)	0.07	(0.34)
Missing			1.77***	(0.32)	1.90***	(0.32)
CCI^a^						
2			0.04	(0.13)	0.10	(0.13)
3			0.21	(0.12)	0.27*	(0.12)
Least severe			− 0.45***	(0.12)	− 0.18	(0.12)
SIMD^a^						
2			0.04	(0.14)	0.05	(0.14)
3			− 0.18	(0.15)	− 0.18	(0.15)
4			0.13	(0.15)	0.16	(0.15)
Least deprived			0.20	(0.13)	0.22	(0.13)
LA^a^						
Clackmannanshire			− 1.12***	(0.30)	− 1.16***	(0.31)
South Ayrshire			− 0.38***	(0.12)	− 0.23	(0.12)
South Lanarkshire			− 0.97***	(0.11)	− 0.95***	(0.11)
Stirling			− 1.39***	(0.20)	− 1.32***	(0.21)
Admission due to injury					0.70***	(0.11)
Long hospital stay					1.30***	(0.08)
Constant	− 8.16***	(0.15)	− 6.17***	(0.41)	− 6.93***	(0.42)
$\sigma _u$	3.09***	(0.08)	2.15***	(0.09)	2.16***	(0.10)
AIC	12,763		11,083		10,753	
BIC	12,791		11,297		10,984	

*$$p<0.05$$, **$$p<0.01$$, ***$$p<0.001$$

^a^The reference category of the age variable is 65–74; the reference category for IORN is the most dependent; for SIMD is the most deprived; for CCI is the most severe;  and the reference category for LA is the City of Edinburgh

**Fig. 1:**
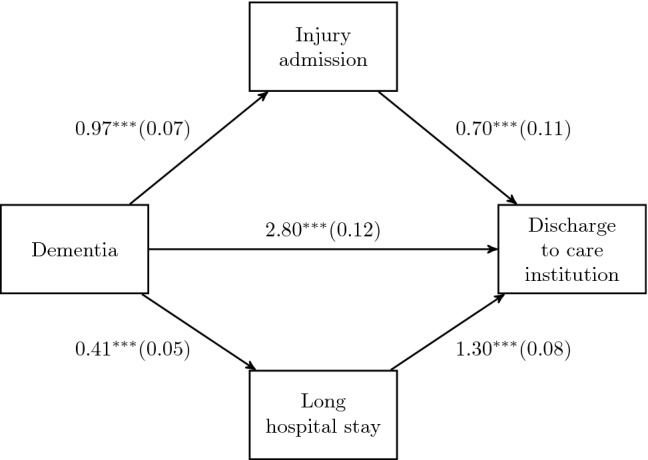
The direct and indirect effects of dementia on discharge to care institutions

We have shown that dementia is a significant predictor of injury-related admissions and long hospital stays. This being the case, we ask whether this will explain, at least partially, why dementia patients are more likely to be discharged to care institutions. Therefore, we have added these two hypothesised mediators to the model. The results are reported in Model III of Table 3. We see that the likelihood of institutionalisation increases if being admitted to hospital due to injury and having a long hospital stay. The coefficient size of the dementia variable becomes smaller after controlling for these two variables. According to the KHB method, this reduction is statistically significant ($z=3.25, p<0.001$). Thus, we conclude that dementia diagnosis has a significant indirect effect on institutionalisation through its impact on injury and long hospital stay. Based on the KHB decomposition, about 6% of the total effect is due to injury and long hospital stay, and the contributions of injury and long hospital stay to the indirect effect are 27% and 73%, respectively. The direct and indirect effects of dementia on institutionalisation are illustrated in Fig. 1.

### The influence of home care

**Table 4:** Results from random effect logistic models on discharge to care institutions, N = 79,983 (43,753)

		Model IV		Model V	
		Coeff.	SE	Coeff.	SE
Dementia patient		3.34***	(0.14)	3.26***	(0.14)
Receive home care in 2010		1.57***	(0.13)	1.31***	(0.14)
Living alone prior to admission		1.08***	(0.21)	0.90***	(0.22)
Admitted from private residence		− 2.67***	(0.10)	− 2.62***	(0.10)
Female		0.22**	(0.08)	0.16	(0.09)
Age^a^					
75–84		0.81***	(0.11)	0.69***	(0.12)
85 or over		2.03***	(0.13)	1.79***	(0.13)
IORN^a^					
*A*–*D*		− 1.03	(0.45)	− 0.97*	(0.45)
0		0.10	(0.33)	0.10	(0.33)
Missing		1.83***	(0.31)	1.95***	(0.31)
CCI^a^					
2		0.06	(0.13)	0.11	(0.13)
3		0.21	(0.12)	0.26*	(0.12)
Least severe		− 0.42	(0.12)	− 0.16	(0.12)
SIMD^a^					
2		0.02	(0.14)	0.03	(0.14)
3		− 0.21	(0.15)	− 0.20	(0.15)
4		0.14	(0.15)	0.16	(0.15)
Least deprived		0.22	(0.13)	0.23	(0.13)
LA^a^					
Clackmannanshire		− 1.09***	(0.30)	− 1.14***	(0.31)
South Ayrshire		− 0.37***	(0.12)	− 0.23	(0.12)
South Lanarkshire		− 0.99***	(0.11)	− 0.96***	(0.11)
Stirling		− 1.40***	(0.20)	− 1.33***	(0.21)
Admission due to injury				0.67***	(0.11)
Long hospital stay				1.28***	(0.08)
Dementia × home care		− 1.81***	(0.20)	− 1.72***	(0.20)
Constant		− 6.32***	(0.40)	− 7.04***	(0.42)
$\sigma _u$		2.12***	(0.10)	2.13***	(0.10)

*$$p<0.05$$, **$$p<0.01$$, ***$$p<0.001$$

^a^The reference category of the age variable is 65–74; the reference category for IORN is the most dependent; for SIMD is the most deprived; for CCI is the most severe; and the reference category for LA is the City of Edinburgh

Now we come to the interpretation of the impact of receiving home care on discharge to care institutions (Table 3). Our estimates seem to suggest that people who receive home care prior to their hospital admissions are more likely to be discharged to institutions. Note that this is unlikely to be due to the confounding effect that people who receive home care may be in poor health conditions or be more dependent as we have already controlled for these potential confounders in our models. The most plausible explanation is that our home care measure is relatively poor. It is not a longitudinal but snapshot measure which was taken in the last week of March 2010. This is right before the observational period of our longitudinal health data, which means that as time passes by, this measure may not truly reflect the home care situation when discharge decisions are made later in the year. To test this, the effect of home care has been evaluated on a sub-sample, including only discharges within a 3-month period since the end of March 2010 when the home care measure is recent and so more likely to be reliable. Using the same model specification, we find no evidence that receiving home care has any influence on institutionalisation based on the sub-sample.

This being understood, our estimates of home care still have valuable implications. As shown in Table 4, we have added an interaction term between dementia and home care to our models. No matter whether admission due to injury and long hospital stay are controlled for or not, the coefficient of this interaction term is highly significant. This indicates that the impact of home care on institutionalisation from hospitals differs for patients with and without dementia. Although receiving home care is associated with increasing odds of institutionalisation for patients without dementia, it decreases the odds for dementia patients.

## Discussion

Although it is well established that a dementia diagnosis is an important predictor of institutionalisation for older people, the underlying mechanisms have rarely been examined. Focusing on the institutionalisation from hospitals, we find that in addition to a direct effect, dementia diagnosis also influences hospital discharge to care institutions indirectly through injury and the length of hospital stay. More specifically, dementia patients are more prone to injury-related hospital admissions. This further explains why they are more likely to be institutionalised as risk of injury could indicate insufficient support to enable independent living at home. Similarly, dementia patients tend to have longer hospital stays, which also partially explains the higher risk of institutionalisation. This is the first contribution of this study.

Secondly, we have examined the association between home care and institutionalisation from a hospital. Due to data constraints, we are not able to say how and to what extent home care influences the risk of institutionalisation for older people, but our results suggest that the impact of receiving home care differs significantly for patients with and without dementia. Adequate support at home is critical in allowing older people to live independently, and these initial findings suggest a particularly important role for home care among older people with dementia.

In this study, our data come from large-scale linked administrative datasets. This gives us an advantage over studies using survey data. The estimated prevalence of dementia among people aged 60 or over is under 6% in Western Europe (Ferri et al. 2005; Prince et al. 2013). Due to the low prevalence, not even mentioning the possibility of under-diagnosis and under-representation, the number of participants living with dementia is often small in surveys that do not explicitly over-sample this group. For instance, in the English Longitudinal Study of Ageing, one of the world leading ageing surveys, there are only less than 1% of older people aged 65 or over recording a dementia diagnosis. Thus, studies using these survey data for dementia research are likely to have limited statistical power for between-group comparisons. Our study also has an advantage over studies using data collected from clinical samples. These data are typically from a specific hospital setting and hence with limited variation. In contrast, our administrative dataset includes every NHS hospital (and NHS contracted beds) across five Scottish local authorities, containing patients with different socioeconomic backgrounds and various health conditions. Furthermore, we are able to link reliable administrative data on the receipt of home care to the health records in order to explore how health and social care services interact. This allows us to make comparisons between patients with and without dementia in the population and to draw a wider inference.

Despite all the merits of the linked health and social care data, they are not without limitations. First of all, neither the health nor social care datasets contain information on whether patients have access to informal care or other formal care services that are not provided (or purchased) by local authorities. Arguably the availability of informal care or other forms of formal care is associated with the usage of home care (Bonsang 2009; Lyons and Zarit 1999; Noelker and Bass 1989; Van Houtven and Norton 2004); and it may also influence institutionalisation for older people (Charles and Sevak 2005; Jette et al. 1995; Lo Sasso and Johnson 2002). To tackle this problem as adequately as the data allow, we have used an indicator of whether patients live alone as a proxy for the availability of informal care. Nevertheless, this proxy measure is not ideal as it does not capture informal care provided by people outside of the patient’s household.

A further concern is the quality of this measure. As it is from the health data, the information may not be recorded accurately when patients were admitted to hospitals. This limitation hinders our efforts to examine directly the relationship between home care and institutionalisation for older people. Future research is needed to take informal care into consideration when relevant data become available and to further investigate the difference between dementia patients and other patients without dementia. Another possible direction for future research is to examine how institutionalisation is affected by the different types and stages of dementia, which has not been investigated in this study due to the lack of information.

In this study, we had an observational period of 1 year using data from five Scottish local authorities. A further extension of these data to include more years, more local authorities, more detailed social care measures, and perhaps even to incorporate some information on informal care, will increase the potential for analyses of this sort to tackle the challenges we face in designing services for later life that adequately meet the needs of ageing populations.
